# A Window to Telehealth Geriatric Social Work Job Market Landscape: A Cross-Sectional Study

**DOI:** 10.1093/geroni/igab046.3042

**Published:** 2021-12-17

**Authors:** Lihua Huang

**Affiliations:** Grand Valley State University, Grand Rapids, Michigan, United States

## Abstract

The purpose of this research study is to explore current Telehealth geriatric social work by describing its most recent job market characteristics. As part of a larger longitudinal research, data for this cross- sectional study was collected in January 2021. Three top job search engines, Google, ZipRecruiter, and Indeed, were used to collect data on Telehealth social work job openings. On each search engine, five searches were completed with the five key words: "social work" Telehealth jobs, LCSW Telehealth jobs, remote LCSW jobs, Telehealth "social work" jobs, and Telehealth "social worker" jobs. It analyzed 112 Telehealth geriatric social work job ads, 12.8% of total 873 ads from these fifteen searches. Results from descriptive and thematic data analysis show large, for-profit organizations are dominating the Telehealth geriatric social work field while small private practices are emerging during the pandemic. The study found Telehealth geriatric social work is providing vital continuum services to older people in communities, hospitals, and long-term care facilities at individual, family, and group levels. The results document innovative technological tools present new methods to engage, assess, and intervene, particularly with mental health needs. The Telehealth organizations are making the pitch to attract competitive professionals. While nearly 1/3 of the sampled organizations stated they intended to make Telehealth/remote positions temporary, the study concludes Telehealth has built its infrastructure and workforce to become an indispensable and ongoing part of gerontological and geriatric social work. Social work education, research, and practice must pay close attention to its implications in skill building.

